# A Review of Environment Effects on Nitrate Accumulation in Leafy Vegetables Grown in Controlled Environments

**DOI:** 10.3390/foods9060732

**Published:** 2020-06-03

**Authors:** Zhonghua Bian, Yu Wang, Xiaoyan Zhang, Tao Li, Steven Grundy, Qichang Yang, Ruifeng Cheng

**Affiliations:** 1 Institute of Environment and Sustainable Development in Agriculture, Chinese Academy of Agricultural Sciences, Beijing 100081, China; zhonghua.bian@ntu.ac.uk (Z.B.); litao06@caas.cn (T.L.); yangqichang@caas.cn (Q.Y.); 2 School of Animal, Rural and Environmental Sciences, Nottingham Trent University, Nottingham NG25 0QF, UK; wangyu985@yeah.net (Y.W.); steven.grundy@ntu.ac.uk (S.G.); 3 Institute of Industrial Crops, Jiangsu Academy of Agricultural Sciences, Nanjing 210014, China; myfair@yeah.net; 4 Institute of Urban Agriculture, Chinese Academy of Agricultural Sciences, Chengdu 610213, China

**Keywords:** nitrate reduction, HY5, light, temperature, CO_2_, fertilizer strategy, water management

## Abstract

Excessive accumulation of nitrates in vegetables is a common issue that poses a potential threat to human health. The absorption, translocation, and assimilation of nitrates in vegetables are tightly regulated by the interaction of internal cues (expression of related genes and enzyme activities) and external environmental factors. In addition to global food security, food nutritional quality is recognized as being of strategic importance by most governments and other agencies. Therefore, the identification and development of sustainable, innovative, and inexpensive approaches for increasing vegetable production and concomitantly reducing nitrate concentration are extremely important. Under controlled environmental conditions, optimal fertilizer/nutrient element management and environmental regulation play vital roles in producing vegetables with low nitrate content. In this review, we present some of the recent findings concerning the effects of environmental factors (e.g., light, temperature, and CO_2_) and fertilizer/nutrient solution management strategies on nitrate reduction in vegetables grown under controlled environments and discuss the possible molecular mechanisms. We also highlight several perspectives for future research to optimize the yield and nutrition quality of leafy vegetables grown in controlled environments.

## 1. Introduction

Nitrogen is a key element for the biosynthesis of nucleic acids, protein, and chlorophyll in plants. Among the various nitrogen sources, nitrate is largely taken up from the soil by plant roots [1]. To ensure the yield of marketable production, nitrate fertilizer is widely applied to crops [2,3]. Due to the increasing demand for inexpensive food and the relatively low cost of mineral fertilizer, overfertilization of nitrogen, especially nitrate fertilizer, has been a common issue in crop production [2,4]. Overfertilization of nitrate not only leads to low nitrogen-use efficiency and accelerates both the eutrophication of water and the acidification of soil but also results in excessive accumulation of nitrates in edible portions of crop plants.

Vegetables play a vital role in the human diet because they are rich in a wide range of beneficial compounds, including vitamins, minerals, and secondary metabolites [5]. However, vegetables, especially leafy vegetables, can accumulate extremely high levels of nitrates (usually nitrate level ≥ 700 mg kg^−1^) during cultivation [6,7]. Although several recent studies have indicated that a short-term high-nitrate diet can alleviate the risk of high blood pressure and cardiovascular disease in some people older than 60 years [8,9], an increasing number of studies have reported that the consumption of vegetables containing a high nitrate concentration every day poses a threat to human health and can cause gastric cancer and methemoglobinemia in infants and children [10,11]. To protect human health, the Joint FAO/WHO Expert Committee suggests that the acceptable and safe daily consumption of nitrate ions should not exceed 0.07 mg kg^–1^ bodyweight day^–1^ [12]. It has been reported that nitrate intake from vegetables accounts for approximately 80% of daily intake in the human diet [13]. Therefore, it is essential for food safety to maintain nitrate levels in vegetables below legal limits. In recent years, the regulation of nitrate concentration in vegetables has generated great concern among researchers and farmers [5,6,14,15,16].

In plants, nitrate accumulation depends on its absorption and metabolism. Some of the root-absorbed nitrates are assimilated in the roots, but most are transported to the shoots and assimilated by nitrate reductase (NR) and other nitrogen metabolism enzymes in plant leaves [17]. The uptake, assimilation, and translocation of nitrates in plants are regulated by multiple internal cues (expression of related genes and enzyme activities) and also by external environmental factors [7]. In addition to better fertilization management strategies, nitrate concentration in vegetables can be reduced through the regulation of the growth environment, thereby promoting the reduction of nitrate into organic nitrogen compounds [5,7,18]. Controlled environmental agriculture, or protected horticulture, as it is also known, has been the most prevalent form of horticultural crop production due to its high yield [19]. Greenhouses are the most widely used protected facilities because they create an ideal environment (e.g., light conditions and temperature) for vegetable production. There are approximately 115 countries in the world commercially producing vegetables using greenhouses, and the total area of worldwide greenhouse vegetable production was 473,466 hectares in 2016 [19]. Combined with soilless cultivation techniques (e.g., hydroponics and aeroponics), protected horticulture has been recognized as a promising method for increasing yields in commercial vegetable production [20]. However, excessive nitrate accumulation in vegetables produced under protected cultivation is also a common issue due to unsuitable nitrate fertilization and/or environmental management strategies [21,22].

In recent years, several reviews have summarized the broader aspects of the effects of nitrogen management on nitrogen-use efficiency and nitrate accumulation in crops [2,21,22,23]. However, reviews concerning the management strategies of nitrate concentration in vegetables produced in controlled environments are relatively limited. Therefore, this review aims to present the current research on decreasing the nitrate concentration in vegetables grown in controlled environments. We also discuss some possible mechanisms concerning nitrate concentration regulation in vegetables and proposed perspectives for future research.

## 2. Nitrate Assimilation and Accumulation in Vegetables in Response to Light Conditions

**Figure 1:**
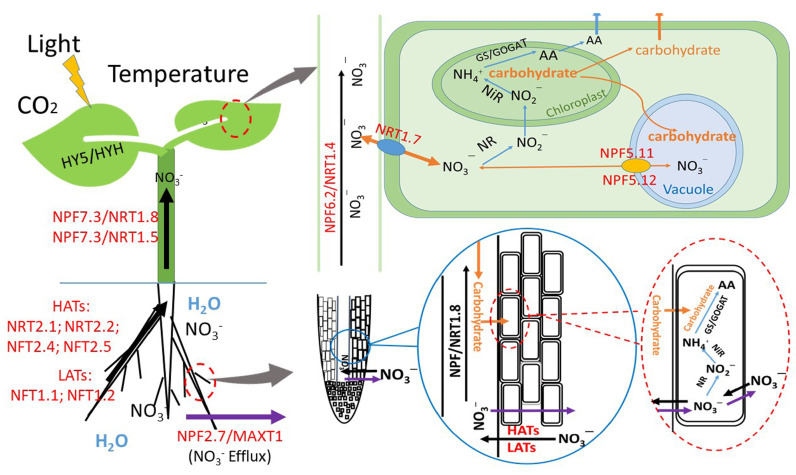
Schematic representation of nitrate absorption, distribution, and assimilation in plants and the function of environmental factors. Light conditions, temperature, and CO_2_ affect nitrate uptake, distribution, and assimilation in plants [7]. HATs, high-affinity transport system. LATs, low-affinity transport system. NRT1 and NRT2 family members are involved in nitrate uptake and transport [24,30,31]. NR and NiR, nitrate reductase and nitrite reductase, respectively; GS/GOGAT, glutamine synthetase/glutamine-2-oxoglutarate aminotransferase; AA, amino acid. “
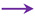
” represents NO_3_^–^ efflux; “
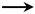
” represents NO_3_^–^ influx and transport in plant roots and stems. “
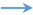
” indicates NO_3_^–^ metabolism. “
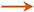
” indicates carbohydrate transfer; “
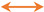
” represents NO_3_^–^ transfer among plant cells.

**Figure 2:**
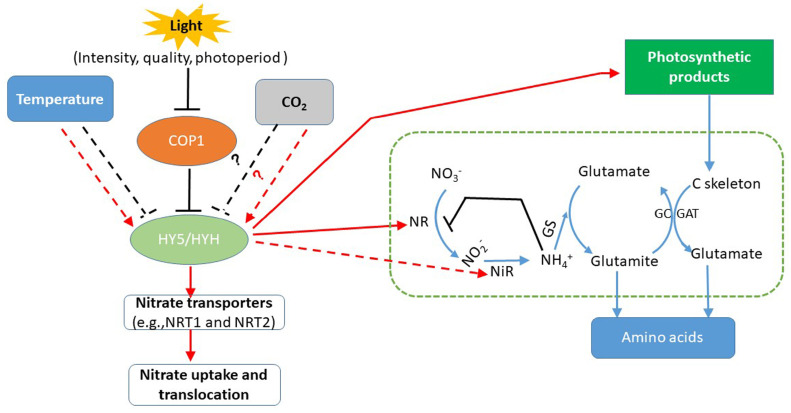
A simplified model of the environmental regulation of nitrate absorption and assimilation in plants. HY5/HYH, the basic leucine (bZIP) transcription factor HYH ELONGATED HYPOCOTYL 5 and its homolog. COP1, the ubiquitin E3 ligase CONSTITUTIVE PHOTOMORPHOGENIC 1, which is involved in the light-related control of nitrate transport and assimilation through interactions with HY5/ HYH [32]. CO_2_, cold and heat stress can exert effects together with the light response via the HY5/HYH pathway [31,33,34], but the mechanism through which temperature stress or other environmental factors, such as CO_2_, regulate nitrate transportation and assimilation is unclear. “
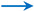
” represents nitrate assimilation under the reduction of nitrogen-metabolizing enzymes. “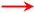 and 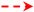
” indicates defined and presumed positive regulation. “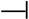 and 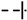
” represents defined and presumed inhibition, whereas “?” indicates a speculation.

Nitrate assimilation in plants starts with the reduction of nitrate to nitrite by NR in the cytoplasm. The resulting nitrite is reduced to ammonium by nitrite reductase (NiR) localised in chloroplasts or in plastids in the roots [24] (Figure 1). Light is one of the main factors regulating plant growth and development (Figure 2), as light not only provides energy for driving photosynthesis but also serves as a transduction signal for triggering the expression of related genes. Light stimulates nitrate reduction by inducing NR-related gene (*NR*) expression, increasing NR activity, and providing available reductants [25]. In plant leaves, the reductants used for nitrate assimilation are produced from photosynthetic electron transport [26], whereas in the roots, these reductants stem from the process of mitochondrial respiration and the pathways of malate and pentose phosphate [27]. After being received by the photosynthetic antenna complex in plant leaves, the absorbed light energy is transferred and stored as ATP and NADPH by the electron and proton transfer complex. Apart from primarily being used for carbon dioxide (CO_2_) fixation, approximately 25% of the resulting ATP and NADPH will be used for nitrate assimilation in plants [28,29]. Thus, excessive accumulation of nitrates in plant tissues frequently occurs under poor light conditions [23].

Supplementary light by artificial light sources is widely used to regulate plant growth, phytochemical concentrations, and nitrate concentration in vegetables produced in controlled environments [6,14,16]. Following the rapid development of light-emitting diode (LED) technology, LEDs have become a viable and innovative alternative to conventional horticultural lighting because LEDs offer unique advantages such as a high energy-use efficiency, long lifespan (approximately 10,000 h), lower heat-put, and flexible spectral control [6]. The application of LEDs as either a sole source of lighting (e.g., closed-type plant factories and growth chambers) or as supplemental lighting, whether alone or mixed with conventional light sources (e.g., fluorescent and high-pressure sodium lamps), enables horticulturists and farmers to regulate the nitrate concentration in vegetables through light condition management in protected facilities [6]. In general, the effects of light on the uptake, assimilation, and distribution of nitrates can be categorized as related to light intensity, duration, or spectra (Figure 2).

### 2.1. The Effects of Light Intensity

Light intensity has profound effects on the nutritional quality of vegetables. In greenhouses, the amount of daylight received can be reduced by 30% or more due to the occlusion of the greenhouse structure. Low light intensity is the primary limiting environmental factor for vegetable growth and nutrient improvement in the greenhouse during winter. This is also a problem during the summer period due to the overcast skies [35]. Increasing light intensity within a certain range can induce the activity of nitrate metabolism enzymes and provide more energy for nitrate assimilation [36]. For instance, under different nitrate supplementation conditions, the nitrate concentration in lettuce (*Lactuca sativa* var. butterhead) leaves significantly decreased when the light intensity of red and blue LED light (red:blue = 4:1) increased from 60 to 210 μmol m^−2^ s^−1^ [37]. Before harvest, supplemental blue LED light increased the growth and nutrients of pak choi (*Brassica rapa*) grown in the greenhouse, while the nitrate concentration decreased as the blue light intensity increased from 0 to 150 μmol m^−2^ s^−1^ [38]. Samuolienė et al. [39] used mixed LEDs (those emitting at 455, 638, 665 and 731 nm) with different light intensities to treat *Brassica* microgreens. The results showed that the nitrate concentration in the microgreens decreased by 37.7%–84.5% with increasing light intensity from 110 to 545 μmol m^−2^ s^−1^. Perez-Lopez et al. [40] studied the interacting effects of light intensity and CO_2_ concentration on the phytochemical compounds of lettuce grown in a growth chamber. Under an ambient CO_2_ atmosphere (approximately 400 μmol mol^−1^), the nitrate concentration in green-leaf and red-leaf lettuce markedly decreased by 25% and 86%, respectively, while the soluble sugar concentration increased by 241% and 41% when light intensity increased from 400 to 700 μmol m^−2^ s^−1^. Additionally, the researchers found that elevated CO_2_ levels enhanced the positive effects of light intensity on nitrate reduction in lettuce. This may have occurred because the regulatory effects of light intensity-induced *NR* expression are closely linked to photosynthetic products [25]. It has been reported that the transcripts of *NR* in *Arabidopsis* seedlings under low light intensity are comparable to those under high light intensity when sucrose is present in the growth medium, but sucrose did not induce *NR* expression under darkness [41]. Furthermore, the increased carbohydrates under high light intensity and elevated CO_2_ can act as carbon skeletons for ammonium (NH_4_^+^) assimilation via the Gln synthetase-Glu synthase cycle (Figure 2), thereby reducing the negative feedback regulation of NH_4_^+^ accumulation to nitrate assimilation in higher plants [42].

The basic leucine zipper (bZIP) transcription factors ELONGATED HYPOCOTYL 5 (HY5) and HY5 HOMOLOG (HYH) are central regulators of nitrate assimilation in plants [32]. The expression of *HY5* is light-intensity-dependent, and high light intensity promotes HY5 accumulation [33]. Previous studies have shown that HY5 positively regulates light-induced NR activity and coordinates nitrogen and carbon acquisition by affecting NR and nitrate uptake-related gene expression [43,44]. Furthermore, under low-light-intensity conditions, the shortage of sucrose impairs HY5-induced *NR* expression in plants, and, in turn, limits nitrate reduction in plant leaves [45]. In addition, there is no advantage for nitrate assimilation in plant leaves under low-light conditions because of the competition of reductants between CO_2_ fixation and nitrate reduction [46]. In addition to directly serving as a nutrient source, nitrates can also function as osmoticums under low-light conditions, leading to excessive nitrate accumulation in plants [47]. Overall, in controlled-environment growth facilities, better light intensity regulation using artificial light sources could pave a new way to producing leafy vegetables with low nitrate concentration. However, further investigations concerning the molecular mechanism of light intensity in the regulation of nitrate assimilation under controlled environments are still needed.

### 2.2. The Function of Light Duration or Photoperiod

Photoperiod or light duration is another vital environmental factor regulating plant flowering, morphogenesis, and metabolism. The extension of light duration or modification of the photoperiod using artificial light sources can increase carbohydrate synthesis, which stimulates nitrate assimilation by providing a continuous supply of reductant energy and carbon skeletons [48]. After being taken up from the soil and transported into different organs, nitrates are generally stored in the vacuole in the dark [49]. During the daytime or under light conditions, nitrates stored in the vacuole are exported to the cytosol via members of the nitrate transporter 1 (NRT1)/peptide transporter (PTR) family (NPF), including NPF5.12 and NPF1.2, and are reduced by NR [50,51]. Thus, harvesting at the end of the light period has been recommended as a way of producing vegetables with low nitrate concentration [52]. When lettuce was grown under red (638 nm) light via LEDs under a day/night temperature of 21/17 °C and light intensity of 150–160 μmol m^−2^ s^−1^, continuous light (CL) for 22 days was beneficial for decreasing the nitrate concentration in ‘Solvano’ baby leaf lettuce, whereas it led to excessive nitrate accumulation in ‘Multired 2’ and ‘Multired 4’ red-leaf lettuce [52]. Zhou et al. [53] reported that CL for 48 h using a mixture of red and blue LEDs (red:blue = 4:1) with a photosynthetic photon flux density (PPFD) of 200 μmol m^−2^ s^−1^ significantly reduced the nitrate concentration in hydroponic lettuce. This may partially be attributed to the fact that CL promotes stable HY5 protein accumulation and activation [33]. The presence of HY5 activates the expression of NR-rated genes and concomitantly increases NR activity to promote nitrate assimilation in plants under CL [54]. However, Bian et al. [6] found that when hydroponic lettuce was grown under CL delivered by different spectral compositions of LEDs, the nitrate concentration in the lettuce leaves cyclically fluctuated, and the lowest nitrate concentration was observed after CL for 24 h. When the duration of CL exceeded 24 h, CL led to a decrease in NR activity and downregulation of *NR* and *NiR* expression, which resulted in a decrease in nitrate reduction and, in turn, caused a re-accumulation of nitrates in the lettuce leaves [6]. These results indicate that other mechanisms may be involved in the regulation of nitrate assimilation in vegetables under CL conditions. Jonassen et al. [55] demonstrated that the expression of *NR* but not other nitrate assimilation-related genes was activated by HY5 and HYH. Under long-term CL, these products of HY5/HYH-induced NR, such as nitrite, nitric oxide, and reactive oxygen species, are toxic to nitrate assimilation as a consequence of the negative feedback on the transcription of *NR* and activity of NR [56,57] (Figure 2). In addition, the shoot-to-root-translocated HY5 under CL can induce *NRT2.1* expression to facilitate nitrate uptake [44], which, to some extent, aggravates nitrate accumulation in plant leaves exposed to long-term CL. In summary, the regulation of the photoperiod or light duration using artificial light sources can be an effective strategy to reduce the nitrate concentration in vegetables. However, in terms of reducing nitrate concentration in vegetables in the greenhouse or closed-type plant factories through light duration management, the vegetable species and other light parameters (light intensity and spectra) should also be considered.

### 2.3. The Function of Light Spectra

Compared with light intensity and light duration, the effects of light spectra on nitrate assimilation in plants are more complex (Table 1). NR is the rate-limiting enzyme that catalyzes the first step of nitrate assimilation. The activity of NR and expression of *NR* are sensitive to light spectra [58,59].

#### 2.3.1. Red and Blue Light

Among the different light spectra, red (600–700 nm) and blue light (400–500 nm) not only are disproportionately absorbed by foliar pigments to drive photosynthesis but also are perceived by photoreceptors to regulate metabolism in plants by triggering downstream gene expression [66,67]. Thus, red and blue wavelengths have been considered as the most efficient light sources for vegetable production in protected facilities. Compared with blue light, red light shows higher efficiency at inducing NR activity and reducing nitrate concentration in higher plants [68]. Similar effects of red-light-induced NR activity have been reported in etiolated barley and maize leaves [69]. Furthermore, preharvest supplementation of red LED light (638 nm) for 3 days significantly reduces nitrate concentration and concomitantly enhances concentrations of secondary metabolites and carbohydrates in different leafy vegetables produced in the greenhouse [64]. These results suggest that a red-light-dependent increase in NR activity may be common among plants. The red light regulation of NR activity is currently believed to be mediated by phytochrome photoreceptors through transcriptional and posttranslational regulation of NR [59]. HY5/HYH is necessary for high *NR* expression under red and far-red light, and PhyA and PhyB play pivotal roles in red- and far-red light-induced *NR* expression and NR activity [41]. Recent studies have explored the molecular mechanism of the red-light-mediated activation of *NR* expression. The regulatory cascade of phyA/phyB-COP1-HY5/HYH is thought to be involved in red-light-induced nitrate assimilation through the regulation of NR activity and *NR* expression [59]. In plants, nitrate reduction is also regulated by *NR* at the posttranslational level. Red light signaling can reverse the dephosphorylation of NR, thereby promoting nitrate assimilation in plant leaves [70]. However, the mechanism of phytochrome-mediated signaling in the regulation of this reversible process remains unknown.

The effects of blue light on nitrate uptake and utilization in plants appear to be much weaker than those of red light. Monochromatic blue light is known to increase the overall nitrogen concentration in plants and promote the distribution of nitrates to plant leaves, which leads to excessive accumulation of nitrate in radish leaves compared with the accumulation in plants exposed to red light [58]. However, when combined with other light spectra, blue light shows positive effects on decreasing nitrate concentration and concomitantly enhances phytochemical accumulation in vegetables. Compared with white LED light only, adding blue LED light to white light was more effective at reducing the nitrate concentration in hydroponic lettuce [62]. These results are consistent with the results of Qi et al. [71], who found that blue fluorescent light significantly reduced the nitrate concentration in spinach (*Spinacia oleracea* L). It is well known that nitrate assimilation has a strong relationship with the photosynthetic capability of plant leaves [25] and that carbohydrates stimulate nitrate assimilation by providing reductant energy and carbon skeletons [7] (Figure 2). Under the same light intensity (210 μmol m^−2^ s^–1^) and photoperiod (12 h) conditions, a combination of red, blue, and white LED light was more effective at reducing the nitrate concentration in lettuce grown in a hydroponic system [16]. This phenomenon was presumed to occur because the broad spectral energy of the red light and blue light in the mixed red, blue and white light contributed to nitrate assimilation in the lettuce plants. According to Osterlund et al. [72], cryptochromes, which are blue light receptors, are involved in nitrate assimilation through the regulation of the COP1-dependent degradation of HY5 upon illumination with blue light. Phytochromes, which can weakly absorb blue light in addition to red and far-red light, may also be involved in blue-light-mediated NR activity and *NR* expression [73]. Glutamine synthetase (GS) is another core enzyme of nitrogen metabolism in plants. Blue light was reported to be involved in the regulation of GS-related gene (*GS*) expression at the transcriptional and translational levels via phytochromes [74]. Therefore, when combined with other light spectra, blue light may enhance nitrate assimilation by upregulating *NR* and *GS* transcriptions and concomitantly enhancing NR and GS activities through all or part of the phytochrome-mediated signaling pathway [59,74]. However, until now, the molecular mechanisms by which blue light is involved in the regulation of nitrate assimilation are still not fully understood.

#### 2.3.2. Green Light, UV Light, and Far-Red Light

In recent years, increasing numbers of studies have shown that other light spectra, including those of green light and UV light, have profound effects on mediating photosynthesis, phytochemical anabolism, and nitrate assimilation in plants [6,7,8]. Green light has positive functions in promoting nitrate reduction in plants when supplemented with other light spectra or natural light (Table 1). For example, the supplementation of high-pressure sodium lamps (170 μmol m^−2^ s^−1^) with 30 μmol m^−2^ s^−1^ green (505 nm) LED light markedly reduces the nitrate concentration and concomitantly enhances the carbohydrate accumulation in lettuce grown in the greenhouse [63]. Similar results were also reported by Chen et al. [62]. With the widespread application of artificial lighting in protected cultivation, CL has been identified as a potential method to increase crop production as well as an effective way to shorten the crop breeding period [75,76]. However, long-term CL induces photodamage and leads to decreases in both crop yield and nutritional quality [77]. Recent studies have shown that adding green light to red and blue LED light can alleviate CL-induced photodamage by upregulating *PsbA* expression to facilitate the *de novo* synthesis of the D1 protein and enhance nitrate assimilation in hydroponic lettuce by increasing NR activity and triggering the upregulation of *NR* expression [14,78]. Based on these results, it is assumed that green light is involved in the regulation of nitrogen metabolism and enhances the plant abiotic stress tolerance of plants. This view is further supported by a recent study by Bian et al. [79]. However, the underlying molecular mechanisms of green light inducing NR activity and *NR* expression in vegetables are still unknown.

Unlike green LED light, far-red light has been shown to induce the dephosphorylation of NR through phytochrome-mediated signaling, which results in the inactivation of NR [70]. Consistent with this, the addition of far-red (735 nm) LED light to cool-white fluorescent lamps leads to a 10-fold excessive nitrate accumulation in spinach plants (*Spinacia oleracea* L. ‘Whitney’) compared with that in plants grown under cool-white fluorescent lamps only [65]. Similarly, yellow (596 nm) and infrared (850 nm) LED light supplementation with white LED light led to excessive nitrate concentration in hydroponic lettuce [62]. Because of the occlusion of the covering material, the composition and intensity of UV light (200–400 nm) in the greenhouse are significantly different from those under outdoor conditions. UV light, especially UV-B (280–315 nm) and UV-A (315–400 nm) light, has generated great concern by researchers due to its unique function in plant photomorphogenesis and phytochemical metabolism [79,80]. However, there is fierce controversy regarding the effects of UV light on plant photosynthesis, growth, and nitrate reduction [80,81,82,83]. UV-A (315–400 nm) was reported to reduce the nitrate concentration in hydroponic lettuce [60]. Brazaitytė et al. [61] used UV-A with three different wavelengths (366, 390, and 402 nm) combined with two irradiation levels (6.2 and 12.4 µmol m^−2^ s^−1^) to treat microgreens. The researchers found that the effects of UV-A on the nitrate concentration in the microgreens varied among species and were mediated by the wavelengths and light intensities of UV-A. In addition, previous studies have suggested UV-B exposure has damaging effects on the photosynthetic capability of a range of species [84,85]. Excessive UV-B light exposure could induce a reduction in the maximum photochemical efficiency of PSII, leading to decreases in the synthesis of NADPH and ATP, which limits the photosynthetic energy used for nitrate reduction [86,87]. UV-B exposure was reported to limit nitrate assimilation, which may be a consequence of UV-B-induced inhibition of NR activity and impairment of the absorption or distribution of nitrate in plants [88,89]. In *Spirodela polyrhiza,* UV-B exposure led to the downregulation of *NR* expression as well as marked decreases in the activities and contents of NR, NiR, GS, and glutamate synthase (GOGAT) [90]. No direct evidence exists showing that UV-B is directly involved in nitrate uptake or distribution, but UV-B can activate *HY5* expression in plants [91]. However, the UV-B photoreceptor UVR8 was shown to increase the stability of COP1 by interacting with the COP-SPA1 complex, which leads to the destabilization of the HY5 protein [92]. These results suggest that UV-B has the opposite effect on the accumulation of *HY5* transcription and HY5 protein stabilization. These results indicate that the negative effect of UV-B exposure on nitrate assimilation occurs at the transcriptional and posttranscriptional levels as well as at the protein level. However, it is still unclear how UV-B irradiation exerts its effects on HY5-regulated nitrate uptake and assimilation in vegetables.

In summary, the differences in light spectra on reducing the nitrate concentration may lie in the fact that the uptake, assimilation, and distribution of nitrate under different light spectra involve complex regulatory networks, which include both transcriptional and posttranscriptional mechanisms of photosynthesis and nitrate assimilation [44,93]. Additional studies are still needed to clarify the molecular mechanisms of light spectra on nitrate uptake and utilization in vegetables.

## 3. Nitrate Uptake, Distribution, and Assimilation in Response to Growth Temperature

Temperature is another important environmental factor for plant growth, development, and reproduction. The effects of temperature on plants can be categorized into air temperature and soil temperature. An optimum temperature can not only enhance photosynthetic capability by regulating the key enzyme activities of CO_2_ fixation but also profoundly affect nitrate uptake, distribution, and assimilation in plants [94] (Figure 1). Greenhouse-grown plants often encounter extreme conditions from chronic and abrupt heat stress during hot summer months [95] or cold stress during off-season cultivation [96]. Heat stress can sharply decrease NR activity and disrupt nitrate assimilation in plants [97]. For instance, raising root-zone temperature leads to the excessive accumulation of nitrate in hydroponic lettuce [30]. Compared with relatively low soil temperature (10 °C), relatively high soil temperature (18 °C) enhances nitrate absorption and leads to the excessive accumulation of nitrate in the roots of radish [31]. Previous studies have shown that a great portion of absorbed nitrate is assimilated in the roots under low temperatures [34]. Under weak light conditions, low temperature not only led to marked decreases in the activities of NR, GOGAT, and GS but also resulted in a low nitrate accumulation in the leaves of tomato [98]. This indicates that, in addition to the inhibition of nitrate reduction, low temperature impairs nitrate uptake and translocation in plants. The translocation of nitrates from the roots to the shoots is regulated by both environmental and intrinsic factors. NRT1.1, NRT1.2, NRT2.1, and NRT2.2 have been demonstrated to be involved in nitrate uptake from the soil into root cells [99,100], while NRT1.5 and NRT1.9 are responsible for root-to-shoot nitrate transport [24]. The transcription factor HY5 is dynamic in plants and plays a central role in nitrate uptake, translocation, and assimilation. A shoot-to-root mobile HY5 has been shown to coordinate carbon and nitrate acquisition and carbohydrate–photosynthate-induced *NRT2.1* expression and nitrate uptake in plant roots [44]. Cold stress (4 °C) or heat stress (42 °C) for 24 h not only leads to the downregulation of *BjNRT1.1*, *BjNRT1.2,* and *BjNRT1.5* but also results in severe downregulation of *BjNR1*, *BjNR2*, and *BjNiR1* in *Brassica juncea* L., which resulted in dysfunction in nitrate uptake and assimilation [101]. However, it has been reported that both low-temperature treatment and short-term heat shock treatment positively enhance the stabilization of the HY5 protein [102,103]. Therefore, under cold and heat stress, how HY5 exerts its effects on nitrate uptake and assimilation remains unclear. In summary, extreme temperatures not only inhibit plant growth but also lead to excessive nitrate accumulation in plants. A suitable temperature regulation strategy is needed to guarantee the production of vegetables with a low nitrate concentration.

## 4. Nitrate Assimilation and Accumulation in Response to Elevated Carbon Dioxide Concentration

Carbon dioxide (CO_2_) is necessary for photosynthesis. It is predicted that the atmospheric CO_2_ concentration will increase to about 800 μmol mol^–1^ by the end of this century. The response of plants to increased CO_2_ is directly connected with their nitrogen status [104]. Nitrate assimilation is one of the most important physiological responses of plants and the sink size of nitrogen plays a vital role in determining the nitrate assimilation response to elevated CO_2_ [105,106]. Previous studies have reported that compared with nitrogen sink-limitation vegetables, some vegetables which have large nitrogen sink organs showed different responses of nitrate uptake and assimilation to elevated CO_2_ [106,107]. It is reported that increasing CO_2_ levels markedly decreased the leaf nitrate concentration in sweet pepper plants under salt stress [108]. Under elevated CO_2_ and unlimited nitrate supply conditions, the increased synthesis and activity of NR coincides with an increase in photosynthesis induced by elevated CO_2_ [109]. Larios et al. [110] reported that a short-term increase in CO_2_ increased carbohydrate levels and concomitantly reduced the nitrate concentration in cucumber leaves by enhancing NR activity and expression of NR-related genes. Plant photosynthesis and leaf nitrogen sink activity are closely coordinated together. The increase in carbohydrates under elevated CO_2_ could further promote the transcription and posttranslational regulation of *NR*, increasing nitrate assimilation [111]. In addition, the enhanced photosynthesis induced by elevated CO_2_ can provide more carbon skeletons and energy for NH_4_^+^ assimilation. Consequently, nitrogen assimilation would be more efficient in some plants under elevated CO_2_ conditions compared with ambient CO_2_ [105,111].

However, some studies have also shown that elevated CO_2_ leads to a substantial decrease in NR activity and directly inhibits nitrate assimilation in the leaves of a variety of C_3_ plant species, such as wheat and *Arabidopsis* [104,112]. These differences in the effects of elevated CO_2_ on nitrate assimilation may be due to the distinct impact of sink-limitation of nitrogen among species [106]. Nitrate assimilation in plants proceeds only when the availability of reduced ferredoxin exceeds the requirement for NADPH synthesis. Under elevated CO_2_ conditions, when the atmospheric CO_2_ concentration is greater than the saturation point, the amounts of reductant and ATP generated by the photochemical process cannot meet the requirements of CO_2_ fixation [113]. The competition for reductants between nitrates and CO_2_ fixation inhibits nitrate assimilation under elevated CO_2_ conditions [114]. In plants, the transcription factor HY5 coordinates photosynthetic carbon fixation and nitrogen metabolism, which enables homeostatic maintenance of the carbon–nitrogen balance in dynamic environments [44]. To date, there has been no evidence that CO_2_ is directly involved in the regulation of *HY5* expression and the stabilization of the HY5 protein. It is possible to speculate that elevated CO_2_ may affect nitrate uptake and assimilation through involvement in the HY5-mediated regulation of nitrates. Enriching CO_2_ levels is a common strategy for increasing the yield and nutritional quality of vegetables grown in control environments. However, in terms of reducing the nitrate concentration and enhancing yield, CO_2_ enrichment should be combined with other environmental regulatory activities, including temperature and light management, and the variation in nitrogen sink size between vegetable species, thereby balancing nitrate assimilation and CO_2_ fixation in vegetables grown under controlled environments.

## 5. Nitrate Assimilation and Accumulation in Response to Fertilizer Management

Recently, increased demand for organic food with high and natural nutrients has promoted the development of organic agriculture. In contrast to mineral fertilizer that directly provides nitrates, organic fertilizer gradually releases its nitrogen content, which limits the excessive accumulation of nitrate in vegetables [115]. However, the high quality of vegetables in organic agriculture is always at the expense of yield loss and a longer growth period. There is great pressure on agriculture to increase food production to feed a continuously increasing global population and concomitantly protect the environment and conserve natural resources [116]. However, because of the depletion of natural resources, deterioration of environmental conditions and crop yield loss caused by the unpredictability of extreme weather events, achieving simultaneously high nitrate-use efficiency and high crop productivity has become a challenge [117]. These limitations of current agriculture have emphasized the urgent need to develop optimal fertilizer strategies to meet the demands of food security and safety, as well as environmental protection.

In the past few decades, many fertilizer management strategies have been investigated by researchers in terms of reducing the nitrate concentration in vegetables grown in controlled environments. A reduction in nitrate concentrations in nutrient solutions significantly decreased the accumulation of nitrates in hydroponic lettuce [118,119,120]. In addition, reducing the ratio of nitrate to other nitrogen sources or replacing nitrate ions with chlorides led to a 20%–40% decrease in the leaf nitrate concentration of hydroponically cultivated vegetables [121,122]. However, nitrate concentration reduction by a limited nitrate supply often results in yield losses, especially when nitrate starvation is not accompanied by the regulation of other environmental factors that affect plant growth and nitrate assimilation [36,37]. Henriques and Marcelis [123] reported that decreasing light intensity could mitigate the yield loss of lettuce caused by nitrate deprivation. However, the mechanism underlying this phenomenon is still unclear.

In addition to traditional nitrate deprivation, the exogenous application of certain substances has positive effects on decreasing the nitrate concentration of vegetables. Lei et al. [124] reported that the exogenous application of selenium (0.5 µmol L^–1^) significantly decreased nitrate concentration in hydroponic lettuce via enhancing the activities of nitrogen metabolism-related enzymes. Furthermore, foliar nutrition solution with urea (1%) and molybdenum (Mo, 1 mg L^–1^) significantly reduced nitrates in lettuce produced in the spring season, while foliar application of benzyl adenine (5 mg L^—1^) was more efficient in decreasing nitrate concentration in lettuce grown in polytunnels in summer-autumn [125]. Moncada et al. [126] reported that adding Mo (0.5, 1.5, and 3.0 μmol L^–^^1^) to nutrient solutions significantly reduced the nitrate accumulation in lettuce, escarole, and curly endive plants grown in a hydroponic floating system. The positive effect of Mo on nitrate reduction may lie in the fact that Mo is fundamental to the function of NR and consequently mediates nitrate metabolism in plants [127,128]. Similar to Mo, another micronutrient element, iron (Fe), plays a crucial role in nitrate assimilation, as Fe acts as a metal cofactor of enzymes involved in nitrate metabolic pathway. A deficiency of Fe in nutrition solution strongly affected nitrate metabolism by limiting NR activity and downregulating *NR* transcripts in the leaves of cucumber [129]. These results indicate that optimal micronutrient management could be an effective way to produce high-quality vegetables with low nitrate concentration.

In recent years, the use of biostimulants, organic extracts with bioactive molecules such as amino acids and vitamins, has been a common practice in sustainable agriculture [130]. Colla et al. [131] reviewed the positive effects of protein hydrolysates as biostimulants on reducing nitrates in a wide range of vegetable species, such as radish, lettuce, swiss chard, and spinach. Similarly, Amanda et al. [132] reported that foliar applications of a biostimulant (Actiwave^®^, Valagro SpA) at a concentration of 6 mL m^–2^ positively reduced the nitrate concentration in lettuce grown in plastic tunnels. It should be noted that the effect of biostimulants on nitrate metabolism in leafy vegetables is species/various-dependent and is also affected by the dose and the time of application [133]. The positive function of biostimulants on nitrate reduction might be elicited by the downregulation of nitrate transporter genes as well as increasing the transcript levels of related genes involved in the nitrogen metabolism pathway, such as *NR* and *NiR* [134,135].

Although many different strategies have been proven to be effective and have been used in reducing nitrates in vegetables, these current conventional fertilizer management strategies could not fundamentally solve the issue of inefficient use of nitrogen fertilizer by crops, which leads to major economic losses for farmers and causes serious environmental problems. In plants, the excessive accumulation of nitrates is mainly a consequence of an imbalance between nitrate absorption and reduction. Plants absorb more nitrates from the soil than is required for their growth, especially when subjected to overfertilization of nitrates [136]. Precision nitrogen management can pave a new way to reduce agricultural inputs and concomitantly alleviate the negative environmental impact of modern agriculture, which can enhance agricultural sustainability from economic and environmental aspects [137]. These precision fertilizer management strategies should be based on the comprehensive consideration of the cross-talk among nitrate supply, environmental factors, and genetic differences in plant species, which affect the uptake, assimilation, and distribution of nitrates in plants. However, at the current stage, the lack of sensors for real-time, rapid, and accurate determination of nitrate concentration in plants and soil/ nutrition solution has limited the application of precise nitrogen fertilizer management in vegetable production in controlled environments [138].

## 6. Nitrate Uptake, Distribution, and Accumulation in Response to Water Quality and Irrigation Management

Irrigation regimes not only affect the yield but also the uptake, distribution, and accumulation of nitrates in crops grown in controlled environments. For instance, deficit irrigation and overirrigation lead to excessive nitrate accumulation in wild rocket grown in the greenhouse because of the negative effects on NR activity induced by water shortage and root hypoxia caused by excessive water in the rhizosphere [139]. However, Koyama et al. [140] found that appropriate rhizosphere drought stress decreased the nitrate concentration by 18% without impairing the final yield of lettuce through lowering the water level of the nutrition solution in hydroponic systems. These conflicting results may be due to the different growth methods and nitrogen management involved, which affect the absorption and metabolism of nitrates in different species of vegetables.

Because of the scarcity of water resources, treated wastewater and saline water have been used for irrigation in many countries, especially in arid and semiarid areas. The reuse of wastewater and saline water can alleviate the water scarcity of agricultural production but their side effects on crop growth and quality should not be ignored [141,142]. For example, irrigation with treated wastewater led to a significant increase in nitrates in pack choi due to the high concentration of nitrates in the treated wastewater even after treatment with membrane bioreactors [143]. Saline water has negative effects on plant growth, but leads to a decrease in nitrate accumulation in lettuce grown under greenhouse conditions [144]. This may be attributed to the competition of the same anion channel between nitrates and chloride (Cl^–1^) at the site of xylem parenchyma cell membranes [145]. In *Arabidopsis*, nitrates and Cl^–1^ share the same anion channels, which are encoded by *AtNPF2.4* and *AtNPF2.5* (Figure 2). The high Cl^–1^ condition leads to the downregulation of *NPF2.4*, which limits the loading of Cl^–1^ and nitrate into the xylem, thereby restricting the root-to-shoot transfer of both nitrates and Cl^–1^, while the upregulation of *NPF2.5* accelerates nitrates and Cl^–1^ efflux from the roots [146,147]. Taken together, irrigation regimes and water conditions should not be ignored in vegetable production under control environments. An optimal irrigation regime based on water conditions could be an effective way to produce low nitrate vegetables and concomitantly achieve water-saving in vegetable production in controlled environments.

## 7. Conclusions

It is well known that nitrate metabolism and accumulation constitute a complex process that is subject to activities of internal nitrogen metabolism-related enzymes, related gene expression and reductant supply, and external environmental factor regulation (Figure 2). To reduce the nitrate concentration and concomitantly maintain or increase vegetable yields and nutrition quality, different nitrate regulatory strategies should be considered when regulating the nitrate concentration in vegetables. Environmentally controlled facilities such as greenhouses and closed-type plant factories provide great convenience for the regulation of nitrate concentration in vegetables. However, additional detailed studies about the following aspects are still needed in terms of decreasing nitrates in vegetables produced in controlled environments:

(1) Nitrate uptake and assimilation are coordinated with photosynthetic carbon fixation in different dynamic environments [44]. The transcription factor HY5/HYH plays a core role in the complex network of nitrate assimilation and photosynthesis in plants [59]. However, the current knowledge is insufficient, and additional studies are still needed to clarify the molecular mechanism by which HY5/HYH regulates nitrate uptake, distribution, and assimilation under different conditions in protected facilities. An improved understanding of HY5/HYH function and signaling could provide more useful information for improving nitrogen-use efficiency and producing low-nitrate vegetables in controlled environments;

(2) It is well known that suitable light conditions are one of the key environmental factors that ensure a relatively high yield and improve nutritional quality. The effects of light spectra on nitrate uptake, assimilation, and distribution are affected by the concentration of some of the micro-elements (e.g., selenium and Cl^–1^) in the nutrition solution/soil and by other environmental factors. For instance, an optimal percentage of red light in light spectra (also known as light recipes) combined with exogenous application of selenium can produce selenium-enriched lettuce with low nitrate concentration [148]. Therefore, when combined with other suitable environmental factors (e.g., micro-elements, temperature, and CO_2_), an optimal light recipe provided by LED light can achieve targeted regulation of nutritional quality and yield of vegetables;

(3) Overfertilization with nitrate is the main reason for excessive nitrate accumulation in crops and other related environmental pollution. Precision management of nutrient elements based on plant growth requirements can pave a new way to increase crop yield, nutrition quality, and concomitantly increase fertilizer-use efficiency. The rapid development of high-throughput genomic technology has enabled biology to enter the era of large datasets (‘big data’) [149]. Future research regarding machine learning with ‘big data’ technology will provide an opportunity for plant scientists to develop precision nitrate fertilizer strategies according to the nitrate requirement of crops at different growth stages, thereby producing leafy vegetables with a low nitrate concentration in controlled environments.

## Figures and Tables

**Table 1:** Effects of LED light spectra on nitrate metabolism in leafy vegetables.

	Lighting Condition			Vegetable Species or Varieties	Nitrate Metabolic Effects	Reference
	Light Spectra	Light Intensity (μmol m^–^^2^ s^–1^)	Light Duration			
UV—A light (315–402 nm)	UV-A (383–426 nm) + red (599–644 nm) and blue (435–489 nm) light	UV-A LEDs: 30.3 Red LEDs: 111 Blue LEDs: 158.7	18 h light/6 h dark	Lettuce (var. crispa)	Nitrate decreased by 300 mg kg^–1^	[60]
	UV-A (366, 390, and 402 nm), + combination of red (638 and 665 nm), blue (447 nm) and far red (731 nm) light	UV-A LEDs: 6.2 and 12.4, Blue LEDs: 21 Red LEDs: 122 Deep red LEDs: 155 Far red LEDs: 2.2	16 h light/8 h dark	Microgreens of basil (*Ocimum basilicum* L., var. Sweet Genovese), beet (*Beta vulgaris* L., var. Bulls Blood) and red pak choi (var. chinensis, Rubi)	Basil exposed to 6.2 µmol m^–2^ s^–1^ UV-A (366 nm): decreased by around 150 mg kg^–1^; Basil exposed to 12.4 µmol m^–2^ s^–1^ UV-A (366, 390, and 402 nm): increased by 50–100 mg kg^–1^; Beet exposed to 6.2 µmol m^–2^ s^–1^ UV-A (366, 390, and 402 nm): increased by 50–100 mg kg^–1^; Pal choi exposed to 6.2 µmol m^–2^ s^–1^ UV-A (366, 390, and 402 nm): increased by around 50 mg kg^–1^	[61]
Blue LED light (400–492 nm)	Blue light (450 nm), + white (400–700 nm) light	Blue LEDs: 30 White LEDs: 170	16 h light/8 h dark	Lettuce (var. crispa ‘Green Oak Leaf’)	Nitrate in leaves decreased by about 200 mg kg^–1^	[62]
Green LED light (492–577 nm)	Green light (505 or 530 nm) + high pressure sodium lamp (HPL) light	Green LEDs: 30 HPL: 170	16 h light/8 h dark	Baby leaf lettuce: red leaf (var. Multired 4), green leaf (var. Multigreen 3), light green leaf (var. Multiblond 2)	Nitrate in red leaf and light green leaf lettuce decreased by 5666 and 4705 mg kg^–1^ under 505 nm LED, 5452 and 9785 mg kg^–1^ under 530 nm LED light; nitrate increased by 3568 and 249 mg kg^–1^ under 505 and 530 nm LED	[63]
	Green light (522 nm) + white (400–700 nm) light	Green LEDs: 30 White LED: 103	16 h light/8 h dark	Lettuce (var. crispa ‘Green Oak Leaf’)	Nitrate decreased by around 200 mg kg^–1^	[62]
	Green light (530 nm) + combined red (660 nm) and blue (450 nm) light	Green LEDs: 33.33 Blue LEDs: 33.33 Red LEDs: 133.34	Illumination for 24 h before harvest	Lettuce (var. Butterhead)	Upregulated NR related gene expression; increased NR activity; decreased nitrate by 80–111 mg kg^–1^	[6,14]
	Green light (494–565 nm) + combined red (599–644 nm) and Blue (435–489 nm) light	Green LEDs: 44.7 Blue LEDs: 108.3 Red LEDs: 147	18 h light/6 h dark	Lettuce (var. crispa)	Nitrate decreased by 400 mg kg^–1^	[60]
White LED (400–700 nm)	White light (400–700 nm) + combination of red (660 nm) and blue (454 nm) light	Total light intensity: 210	16 h light/8 h dark	Boston lettuce (var. capitate)	Nitrate decreased by 20 mg kg^–1^ (dry weight)	[16]
Red LED (600–700 nm)	Red light (638 nm) + HLP and nature light	Red LEDs + HPL (90) + Nature light intensity = 300	Illumination for 3 days before harvest	Spinach, parsley (*Petroselinum crispum* Mill.), dill (*Anethumgraveolens*)	Reduced nitrate in spinach, parsley, and dill decreased by 206, 566, and 1811 mg kg^–1^, respectively	[64]
Yellow light (577–597 nm)	Yellow light (596 nm) + white light (400–700 nm) light	Yellow LEDs: 30 White LEDs: 103	16 h light/8 h dark	Lettuce (var. crispa ‘Green Oak Leaf)	Nitrate in leaves increased by around 200 mg kg^–1^	[62]
Far-red (735nm and infrared light (850 nm)	Far-red light (735 nm) + cool fluorescent lamp	Far-red LEDs: 86 Cool fluorescent lamp: 187	Illumination for 5 days before harvest	Spinach (var ‘Whitney’).	Nitrate excessive accumulation (0.12 mmoles g^–1^) by 10 times compared with cool fluorescent lamp only (0.01 mmoles g^–1^)	[65]
	Infrared light (850 nm) + White light (400–700 nm)	Infar-red LEDs: 30 White LEDs: 103	16 h light/8 h dark	Lettuce (var.crispa ‘Green Oak Leaf)	Nitrate in leaves increased by about 160 mg kg^–1^	[62]
